# Characterization of a novel multidrug resistance plasmid pSGB23 isolated from *Salmonella enterica* subspecies enterica serovar Saintpaul

**DOI:** 10.1186/s13099-018-0249-6

**Published:** 2018-06-04

**Authors:** Yichen Ding, Ye Htut Zwe, Seow Fong Chin, Gurjeet S. Kohli, Daniela I. Drautz-Moses, Michael Givskov, Jorgen Schlundt, Stephan C. Schuster, Hyun-Gyun Yuk, Liang Yang

**Affiliations:** 1 Singapore Centre for Environmental Life Sciences Engineering (SCELSE), Interdisciplinary Graduate School, Nanyang Technological University, Singapore, Singapore; 2 Singapore Centre for Environmental Life Sciences Engineering (SCELSE), Nanyang Technological University, Singapore, 637551 Singapore; 3 School of Biological Sciences, Nanyang Technological University, Singapore, Singapore; 4 Food Science and Technology Program, Department of Chemistry, National University of Singapore, Singapore, Singapore; 5 Costerton Biofilm Center, Department of Immunology and Microbiology, University of Copenhagen, 2200 Copenhagen N, Denmark; 6 Nanyang Technological University Food Technology Centre, School of Chemical and Biomedical Engineering, Nanyang Technological University, Singapore, Singapore; 7 Department of Food Science and Technology, Korea National University of Transportation, Cheongju, Republic of South Korea

## Abstract

**Background:**

*Salmonella enterica* subspecies enterica serovar Saintpaul (*S*. Saintpaul) is an important gut pathogen which causes salmonellosis worldwide. Although intestinal salmonellosis is usually self-limiting, it can be life-threatening in children, the elderlies and immunocompromised patients. Appropriate antibiotic treatment is therefore required for these patients. However, the efficacy of many antibiotics on *S. enterica* infections has been greatly compromised due to spreading of multidrug resistance (MDR) plasmids, which poses serious threats on public health and needs to be closely monitored. In this study, we sequenced and fully characterized an *S. enterica* MDR plasmid pSGB23 isolated from chicken.

**Results:**

Complete genome sequence analysis revealed that *S.* Saintpaul strain SGB23 harbored a 254 kb megaplasmid pSGB23, which carries 11 antibiotic resistance genes responsible for resistance to 9 classes of antibiotics and quaternary ammonium compounds that are commonly used to disinfect food processing facilities. Furthermore, we found that pSGB23 carries multiple conjugative systems, which allow it to spread into other Enterobacteriaceae spp. by self-conjugation. It also harbors multiple types of replicons and plasmid maintenance and addictive systems, which explains its broad host range and stable inheritance.

**Conclusions:**

We report here a novel MDR plasmid pSGB23 harboured by *S. enterica*. To our knowledge, it carried the greatest number of antibiotic resistance genes with the broadest range of resistance spectrum among *S. enterica* MDR plasmids identified so far. The isolation of pSGB23 from food sources is worrisome, while surveillance on its further spreading will be carried out based on the findings reported in this study.

## Background

*Salmonella enterica* serovar Saintpaul (referred to as *S.* Saintpaul hereafter) is a foodborne gut pathogen and a major cause of salmonellosis worldwide [1]. *S.* Saintpaul was reported to be responsible for several recent large outbreaks of salmonellosis, which affected hundreds of people across multiple cities and states [2, 3]. These outbreaks were mainly due to contamination of food such as unpasteurized fruit juice and raw products, suggesting that food processing facilities are one of the main sources for the dissemination of *S.* Saintpaul [2, 4].

Although intestinal salmonellosis is normally self-limiting and does not require antibiotic treatment in healthy adults, it can be life-threatening among children, the elderlies, and immunocompromised patients [4]. Therefore, appropriate antimicrobial treatments are required for these patients [4]. It was suggested that fluoroquinolones such as ciprofloxacin, the third-generation cephalosporins such as ceftriaxone, and trimethoprim–sulfamethoxazole can be beneficial for patients with *Salmonella* infections [5]. However, the emergence of multidrug resistant *Salmonella* strains worldwide has challenged the effectiveness of these antibiotics [6]. In *Salmonella*, the acquisition of antibiotic resistance genes is usually mediated by MDR plasmids, many of which can spread among *Salmonella* and other Enterobacteriaceae spp. by conjugation [6]. The surveillance on novel MDR plasmids in *Salmonella* is therefore important for the understanding of their epidemiology and transmission, which can provide clues for the design of effective therapies and the control of their further spreading.

In this study, we sequenced and characterized a novel MDR plasmid carried by a *S.* Saintpaul strain isolated from chicken in a food market in Singapore. This plasmid displays a mosaic backbone structure and encodes resistance mechanisms to 9 classes of antibiotics and the commonly used disinfectant quaternary ammonium compounds (QACs). The further spreading of this plasmid among *Salmonella* and other Enterobacteriaceae spp. may pose serious threats to public health.

## Methods

### Conjugation and antibiotic susceptibility test

The *S.* Saintpaul strain SGB23 was previously isolated from a local food market. Conjugation between *S.* Saintpaul SGB23 and the azide-resistant *E. coli* J53 strain was performed on LB agar plates at 25 and 37 °C for 48 h. Briefly, overnight culture of the two strains was washed with 0.9% NaCl for three times. Bacterial suspension of the two strains was mixed at 1:1 ratio with approximately 10^9^ colony forming units (CFU) of each strain and spotted onto a filter paper placed on LB agar plates. Transconjugants were selected using LB agar plates containing 100 μg/ml of sodium azide and 100 μg/ml of ampicillin. Conjugation efficiency was calculated by dividing the CFU of transconjugants by the CFU of the donor strain SGB23. Antimicrobial susceptibility test was performed by broth microdilution using Mueller–Hinton Broth. The inoculum for each strain was approximately 10^6^ CFU/ml, followed by incubation at 37 °C for 18 h.

### Sequencing, assembly, and annotation

The total DNA of SGB23 was purified using Blood and Cell Culture DNA Midi Kit (Qiagen) and sequenced on a PacBio RS II system. The full-length chromosome and plasmid contigs of SGB23 were assembled from long reads obtained from the PacBio RS II system by using HGAP2 pipeline. The low-quality ends of the assembled contigs were trimmed using CLC Genomic Workbench v10.0 with an estimated error rate of lower than 0.1% as the cut-off. The overlapping regions of the trimmed contigs were further identified and resolved by BLASTn search assisted with manual curation. The plasmid sequence was uploaded to the rapid annotations using subsystem technology (RAST) server for initial gene prediction and annotation, followed by manual BLASTp search to ensure accurate annotation [7]. Antibiotic resistance genes carried by pSGB23 were predicted using the ResFinder 2.1 server [8], whereas the IS26 sequences were identified by the ISfinder [9]. Comparative sequence analysis of pSGB23 with pEC2–4 was performed by BLASTn search using BLAST Ring Image Generator 0.95 [10], with antibiotic resistance genes and IS elements labeled in the figure as instructed by the manual.

### Quality assurance

Genomic DNA used for sequencing was isolated from a single colony of SGB23 to avoid contamination caused by allochthonous microorganisms. In addition, PacBio assembly only generated two contigs, which are the chromosome and plasmid sequences of SGB23, whereas no other assemblies were identified. Low-quality regions at both ends of the assembled contigs were trimmed to ensure high-accuracy of the genome as described in “Methods”.

## Results and discussion

In 2016, an MDR *S.* Saintpaul strain SGB23 was isolated from chicken meat in a local food market in Singapore. The whole-genome of SGB23 was sequenced on a PacBio RS II platform and the sequencing reads were successfully assembled by HGAP 2.0 into two contigs: one chromosome contig with 89-fold coverage and one plasmid contig with 109-fold coverage. The close coverages of the chromosome and plasmid sequences suggests that pSGB23 plasmid has a low copy number. The sizes, GC content and coding capacity of SGB23 chromosome and pSGB23 plasmid are summarized in Table 1.

**Table 1:** Length, GC contents and coding capacity of the chromosome and plasmid of *S.* Saintpaul SGB23

	Length (bp)	GC content (%)	Protein-coding genes	tRNA	rRNA
SBG23 chromosome	4,792,385	52.2	4803	85	14
pSGB23 plasmid	254,041	47.5	313	0	0

The coding sequences were predicted and annotated by the RAST server as described in “Methods”

**Fig. 1:**
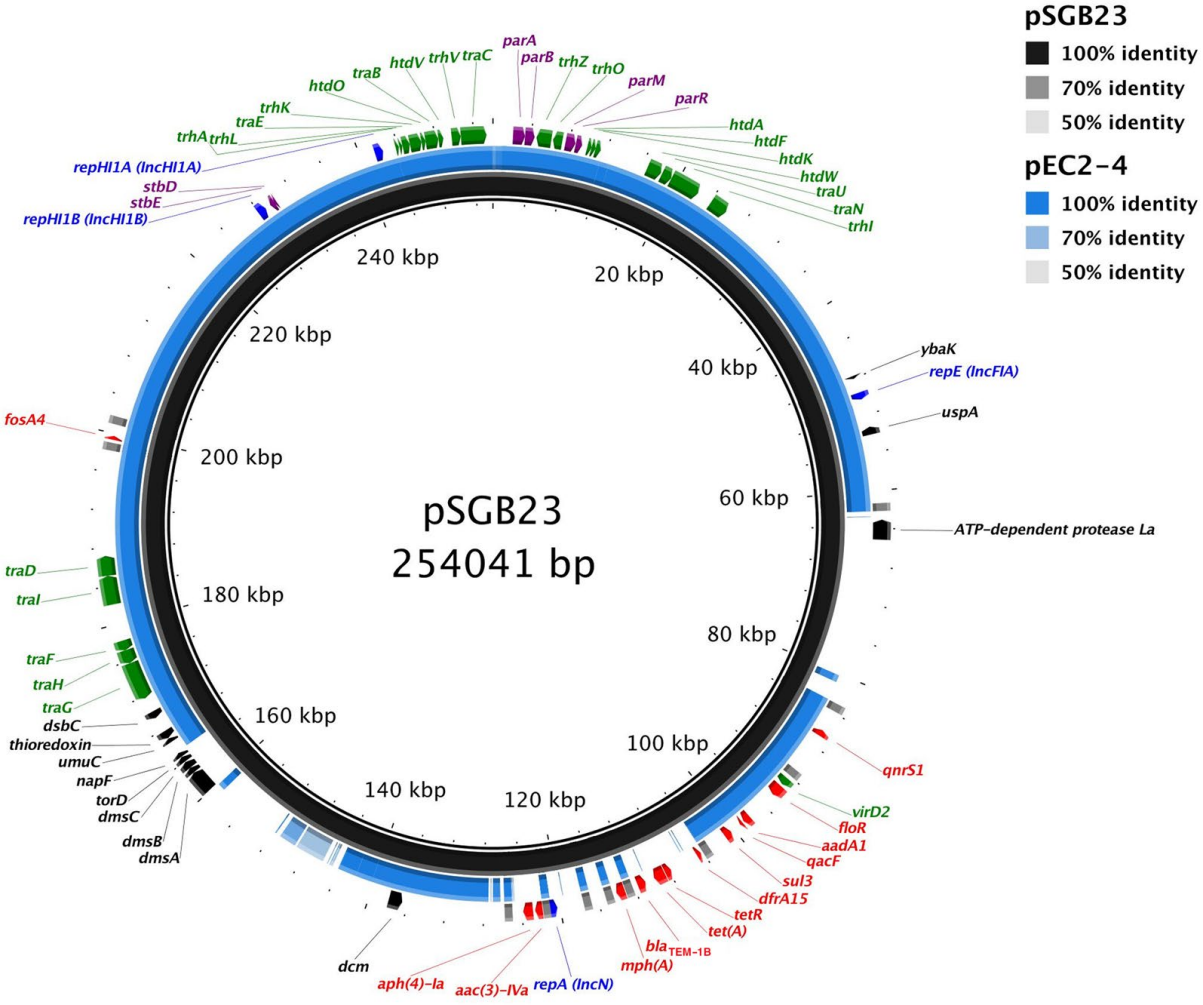
The sequence map of pSGB23 in comparison to pEC4-2 (CP016184). Black circle: pSGB23; blue circle: pEC2–4. The outermost circle shows the predicted genes encoded by pSGB23 with the arrow indicates the direction of transcription. Blue: plasmid replication proteins with their respective Inc types; green: genes related to conjugative transfer; red: antimicrobial resistance genes; purple: genes involved in plasmid partition and addiction systems; grey: IS26 sequences; black: genes with other predicted functions. Other genes encoding proteins with hypothetical functions and transposases are not shown in the figure

To further identify the MDR determinants of *S.* Saintpaul SGB23, we analyzed the acquired antibiotic resistance genes carried by SGB23 using the ResFinder 2.1 server [8]. Interestingly, the chromosome of SGB23 encodes no antibiotic resistance genes, whereas its plasmid pSGB23 is the sole antimicrobial determinant. To understand this epidemically important MDR plasmid pSGB23, we annotated its sequence on the RAST server [7] assisted with manual BLASTp search, analyzed its replicon sequences by using the PlasmidFinder 1.3 server [11]. In addition, we found that the closest sequence to pSGB23 in GenBank is plasmid pEC2–4 (CP016184) [12], which covers 77% of pSGB23 sequence with a similarity of 99%. pEC2–4 is an IncHI1 plasmid recently isolated in Malaysia and carries the colistin resistance gene *mcr*-*1* [12]. The comparison between pSGB23 and pEC2–4 with the predicted replicons and predicted antibiotic resistance genes in pSGB23 are summarized in Fig. 1.

In total, pSGB23 harbours four replicons, which include two IncHI-specific replicons designated RepHI1A and RepHI1B, one IncFIA replicon, and one IncN replicon (Fig. 1). The multiple replicons harbored by pSGB23 may give it competitive advantages over plasmids with single replicon, and therefore, allow it to adopt a broader host range within the Enterobacteriaceae [13, 14]. In particular, the IncFIA replicon was reported to commonly present in IncHI1 plasmids and is responsible for the one-way incompatibility between IncHI1 and IncF plasmids [13].

The IncHI1 type plasmids have been identified from several *S. enterica* serovars and were mainly associated with multidrug or heavy metal resistance [14, 15]. Similarly, we found that pSGB23 carries 11 antibiotic resistance genes, which are responsible for resistance to aminoglycosides (*addA1*, *acc(3)*-*Iva* and *aph(4)*-*Ia*), macrolides (*mph(A)*), quinolones (*qnrS1*), tetracycline (*tet(A)*), β-lactams (*bla*_*TEM*-*1B*_), chloramphenicol and florfenicol (*floR*), fosfomycin (*fosA4*), sulfamethazine (*sul3*) and trimethoprim (*dfrA15*) (Fig. 1). In addition, it also harbors a *qacF* gene responsible for resistance to QACs, which are a class of disinfectants commonly used in food production industry and clinical settings [16]. Most of the antimicrobial resistance genes except for *fosA4* are located in an antibiotic resistance gene (ARG) cluster. Furthermore, the acquisition of antimicrobial resistance genes by pSGB23 probably involves IS26-mediated integration, as 10 of the 11 IS26 elements identified by ISfinder [9] were found to flank the antimicrobial resistance genes (Fig. 1). Interestingly, the IncN replicon is located in the ARG cluster and is also flanked by two IS26 elements, suggesting that it is probably acquired by IS26-mediated integration.

The IncHI1 type plasmids were previously reported to have a thermosensitive mode of conjugation, in which optimal conjugative transfer occurs between 22 and 28 °C, whereas inhibition of conjugation was observed at 37 °C [14]. In pSGB23, we identified 23 conjugative genes, which include 10 *tra* genes, 7 *trh* genes and 6 *htd* genes that are located in two clusters (Fig. 1). To verify if pSGB23 is self-transmissible by conjugation, we performed mating experiments between SGB23 and the azide-resistant recipient strain *Escherichia coli* J53 on solid agar surface for 48 h. The conjugative efficiency of pSGB23 is 3.1 ± 1.4 × 10^−3^ at 25 °C and 3.3 ± 0.8 × 10^−5^ at 37 °C (n = 4), suggesting that pSGB23 is self-conjugative and the conjugation activity is partially inhibited at 37 °C.

We further determined the minimal inhibitory concentrations (MICs) of *E. coli* J53/pSGB23 and *S.* Saintpaul SGB23 to different antimicrobial agents by broth microdilution. It was found that *E. coli* J53/pSGB23 showed increased resistance to 12 antibiotics belonging to 9 different classes, and at-least 2-fold increase in the MICs of two tested QAC compounds cetrimonium bromide and cetylpyridinium chloride compared to *E. coli* J53 (Table 2). This resistance profile is consistent with the antimicrobial resistance genes carried by pSGB23 (Table 2, Fig. 1). Among these tested antibiotics, ampicillin, ceftriaxone, chloramphenicol, ciprofloxacin and trimethoprim–sulfamethoxazole are the first-line drugs to treat severe *Salmonella* infections, whereas azithromycin has been increasingly used to manage both Typhi and non-Typhi *Salmonella* infections that showed poor response to the recommended first-line drugs [17]. Therefore, the spread of pSGB23 to other *S. salmonella* serovars can greatly compromise the efficacies of these antibiotics in treating *Salmonella* infections. In addition, resistance to QACs may allow the host bacteria to better adapt to the food processing facilities, where QACs are extensively used as disinfectants to inhibit microbial growth and prevent foodborne illness [18]. It was also noted that the resistance patterns between *E. coli* J53/pSGB23 and *S.* Saintpaul SGB23 were very similar, which confirmed that pSGB23 is the only resistance determinant of *S.* Saintpaul SGB23.

**Table 2:** MICs of *E. coli* J53/pSGB23 and *S.* Saintpaul SGB23 to various antibiotics (unit: μg/ml)

	*E. coli* J53	*E. coli* J53/pSGB23	*S.* Saintpaul SGB23
Ampicillin	32	> 1024	> 1024
Carbenicillin	16	> 1024	> 1024
Ceftriaxone	1	8	8
Azithromycin	8	32	64
Ciprofloxacin	0.03	0.5	1
Chloramphenicol	16	> 64	> 64
Fosfomycin	1	> 64	> 64
Gentamicin	2	128	128
Streptomycin	8	32	32
Tetracycline	2	64	64
Trimethoprim	0.5	> 256	> 256
Sulfadimethoxine	64	> 256	> 256
Cetrimonium bromide	16	64	64
Cetylpyridinium chloride	8	16	16
Colistin	1	1	2

*E. coli* J53/pSGB23 and *S.* Saintpaul SGB23 shared similar resistance profiles with increased resistance to nine classes of antibiotics and QACs, whereas they remain sensitive to colistin

The large and low-copy-number plasmids such as pSGB23 are usually under the risk of plasmid loss during cell division. Therefore, many of these plasmids have evolved active maintenance mechanisms to ensure their stable inheritance by the daughter cells during cell division [19]. For instance, the plasmid partition systems such as ParA/ParB actively segregate plasmid copies to the daughter cells, whereas the addiction systems such as StbA/StbB act like toxin/antitoxin that eliminates plasmid-free daughter cells [19]. In pSGB23, we found two types of partition systems designated ParA/ParB and ParM/ParR that are embedded among the conjugative genes, and the toxin/antitoxin system StbD/StbE that are at downstream of the RepHI1B replicon (Fig. 1). The presence of plasmid maintenance and addiction systems can ensure the stable inheritance of pSGB23 in the absence of selection pressure posed by antibiotics.

pSGB23 also carries genes that were previously reported to be implicated in stress response, such as *ybaK*, *uspA*, *dsbC*, *umuC* and a gene encoding ATP-dependent protease La, and genes involved in specific metabolic functions such as the *dmsABC* operon, *napF*, and *torD* (Fig. 1). Their functional implications on the host remains unclear.

In summary, we report here a novel plasmid pSGB23 isolated from *S. enterica*. The plasmid harbors both IncHI1 and IncN type replicons and carries 12 antimicrobial resistance genes, which can confer resistance to 9 classes of antibiotics and the QAC class of disinfectants on its hosts. To the best of our knowledge, this is the first report of an IncHI1 plasmid harboring an IncN replicon. pSGB23 also carries the greatest number of antimicrobial resistance genes with the broadest range of resistance spectrum among the *Salmonella* MDR plasmids identified so far. pSGB23 may spread into other Enterobacteriaceae spp. owing to its versatile replication and conjugative systems, as well as its plasmid maintenance and addiction systems. The emergence of pSGB23 may pose serious threats to public health.

## Abbreviations

MDR: multidrug resistant
QACs: quaternary ammonium compounds
ARG: antibiotic resistance gene
*S.* Saintpaul: *Salmonella enterica* serovar Saintpaul
MICs: minimal inhibitory concentrations
CFU: colony forming units
